# Stochastic Model of Integrin-Mediated Signaling and Adhesion Dynamics at the Leading Edges of Migrating Cells

**DOI:** 10.1371/journal.pcbi.1000688

**Published:** 2010-02-26

**Authors:** Murat Cirit, Matej Krajcovic, Colin K. Choi, Erik S. Welf, Alan F. Horwitz, Jason M. Haugh

**Affiliations:** 1 Department of Chemical and Biomolecular Engineering, North Carolina State University, Raleigh, North Carolina, United States of America; 2 Department of Cell Biology, University of Virginia, Charlottesville, Virginia, United States of America; California Institute of Technology, United States of America

## Abstract

Productive cell migration requires the spatiotemporal coordination of cell adhesion, membrane protrusion, and actomyosin-mediated contraction. Integrins, engaged by the extracellular matrix (ECM), nucleate the formation of adhesive contacts at the cell's leading edge(s), and maturation of nascent adhesions to form stable focal adhesions constitutes a functional switch between protrusive and contractile activities. To shed additional light on the coupling between integrin-mediated adhesion and membrane protrusion, we have formulated a quantitative model of leading edge dynamics combining mechanistic and phenomenological elements and studied its features through classical bifurcation analysis and stochastic simulation. The model describes in mathematical terms the feedback loops driving, on the one hand, Rac-mediated membrane protrusion and rapid turnover of nascent adhesions, and on the other, myosin-dependent maturation of adhesions that inhibit protrusion at high ECM density. Our results show that the qualitative behavior of the model is most sensitive to parameters characterizing the influence of stable adhesions and myosin. The major predictions of the model, which we subsequently confirmed, are that persistent leading edge protrusion is optimal at an intermediate ECM density, whereas depletion of myosin IIA relieves the repression of protrusion at higher ECM density.

## Author Summary

Cell migration is fundamental to human physiology and a phenomenon of long-standing interest in cell biology. It requires the concerted regulation of several dynamic processes that mediate physical anchorage of the cell and productive generation of protrusion and traction forces that propel the cell forward. In this work, we have developed a mathematical model that describes this interplay, cast at the level of biochemical signaling pathways activated at the front of a moving cell. Based on our analysis of the model and experimental confirmation of its basic predictions, we assert that coupled, counteracting feedback loops constitute a functional switch between maintenance and stalling of the cell protrusion speed. Our model successfully explains the dependence of this switch on the abundance of adhesive molecules in the cell's immediate surroundings and sheds light on how non-muscle myosin shapes that dependence.

## Introduction

In multicellular organisms, cell migration is of paramount importance for physiological processes such as tissue homeostasis and repair, immune surveillance and response, and developmental patterning. In culture, the crawling of mammalian cells on a surface coated with extracellular matrix (ECM) protein such as fibronectin is classically described as a cycle of distinct subprocesses: membrane protrusion and formation of new adhesive bonds with the underlying substratum at the cell's leading edge, followed by contraction of the cell body forwards, and finally detachment of adhesions at the cell's rear [1]. The primary molecular hubs for the integration of these subprocesses are integrins, adhesion receptors that recognize specific ECM proteins. Upon ligation, integrins cluster to form adhesive contacts that orchestrate the activation of a host of signal transduction pathways and the anchorage of actin filaments inside the cell [2],[3]. Thus, they provide not only physical linkages between the ECM and actin cytoskeleton, through which myosin II motors generate contractile force, but also platforms for localizing biochemical signals that govern leading edge protrusion [4],[5]. Of particular importance in that regard is the integrin-mediated activation of Rac. Its isoforms are small GTPases of the Rho family that, among other cellular functions, promote cell spreading and formation of broad, flat membrane structures called lamellipodia [6]. Despite these molecular insights, the bases for the dynamics of cell migration subprocesses, seemingly stochastic on the one hand, yet spatiotemporally coordinated on the other, are only beginning to be clarified [7].

One of the most mechanistically telling aspects of cell migration is its dependence on ECM density. The general observation is that overall migration speed, determined from the movement of the cell centroid, is optimal at an intermediate ECM density [8],[9]. The physical interpretation of this finding was that the optimal ECM density corresponds to a density of integrin-ECM bonds that allows for both productive motility at the cell front and detachment of older adhesions at the rear of the cell, through myosin-dependent contractility. More recently, this conceptual model has been refined based on detailed measurements of F-actin dynamics and myosin II recruitment in PtK_1_ cells, revealing an optimal myosin II/F-actin density ratio at intermediate ECM density [10].

Further insight came through the implication that not all adhesions actively contribute to membrane protrusion signaling; apparently, only newer (nascent) adhesions formed at the cell's leading edges do [11]. It seems that maturation of a nascent adhesion to form a stable, focal adhesion, marked by actomyosin-dependent growth of the complex perpendicular to the leading edge [12], is accompanied by loss of its ability to mediate protrusion signaling. In Chinese hamster ovary (CHO).K1 cells expressing paxillin-enhanced green fluorescent protein (EGFP), total internal reflection fluorescence (TIRF) microscopy has revealed that, during steady protrusion, the small nascent adhesions are rapidly formed and turned over [13], in proportion to the protrusion velocity [14]. This phenotype is mediated by signaling through Rac, which can be activated in a variety of ways, one of them involving the Rac effector, p21-activated kinase (PAK). Among its various functions, active PAK phosphorylates the focal adhesion protein paxillin on Ser^273^, providing a binding site for the recruitment of the scaffold protein GIT1; GIT1 binds both βPIX, a guanine-nucleotide exchange factor that activates Rac, and PAK, which is activated in turn by Rac. Thus, the pathway constitutes a positive feedback circuit. Disrupting the circuit, for example through expression of paxillin with Ser^273^→Ala mutation or kinase-dead PAK, abrogates protrusion and nascent adhesion formation, whereas expression of paxillin with phosphorylation-mimicked Ser^273^→Asp mutation or constitutively active PAK enhances these responses [13]. Myosin II opposes the influence of Rac/PAK signaling in this context, promotes adhesion maturation, and strongly inhibits the protrusion phenotype [15],[16]; this effect is expected to be more prominent at higher ECM density [17].

Here, through computational modeling and stochastic simulations, we develop new ideas about mechanisms that might give rise to the dynamical interplay between cell protrusion and adhesion at the cell's leading edge(s) [18]–[20]. Analysis of the model suggests that protrusion signaling mediated by nascent adhesions is inherently sensitive because of positive feedback but also susceptible to regulation by other feedback loops involving stable adhesions and myosin II. These regulatory mechanisms shape the dependence of the protrusion/adhesion phenotypic balance on ECM density, which we compare to experimentally observed dynamics in CHO.K1 cells.

## Results

### Computational model of adhesion/protrusion dynamics

**Figure 1:**
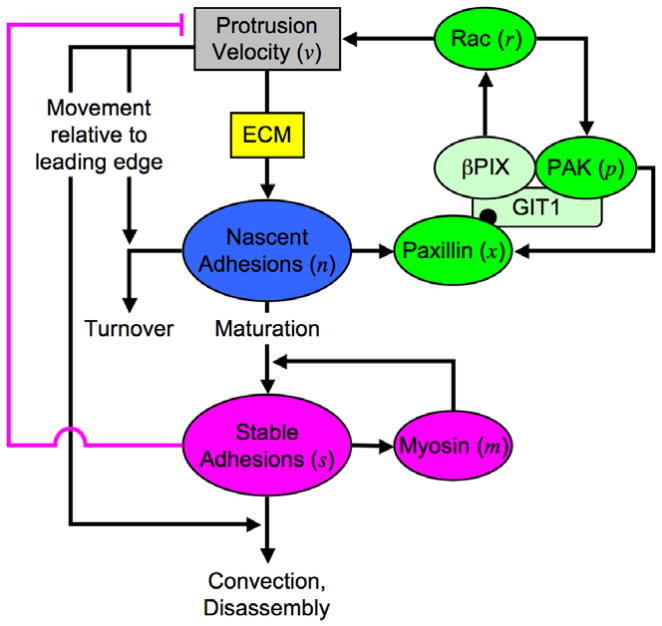
Model schematic. The rates of nascent adhesion formation and turnover depend on the velocity of membrane protrusion (*v*), and the formation rate depends also on the density and composition of ECM. Nascent adhesions promote further protrusion by mediating activation of Rac, utilizing a pathway that is reinforced by positive feedback as shown. Those nascent adhesions that are not turned over mature to form stable adhesions, a process that is reinforced by myosin-mediated feedback in our model. We also include a mechanism whereby stable adhesions directly antagonize protrusion. Stable adhesions disassemble over a relatively long time scale, and their influence on processes at the leading edge is also diminished by convective (*v*-dependent) transport.

We have built a mathematical model with basic relationships among molecular species and processes as illustrated in Fig. 1 (details given under Materials and Methods and in Supplemental Table S1). In our model, nascent adhesions assemble in proportion to membrane protrusion [14]; the rationale is that protrusion is a mechanism for exploring new regions of the substratum and for convective transport of integrins from the top of the cell. In the model, protrusion responds to the activity of Rac in a saturable manner; there is a maximum protrusion velocity attainable. For a given protrusion rate, the efficiency of nascent adhesion formation depends on ECM density, although at high ECM density it is possible that other molecules are limiting for nucleation of adhesions; therefore, the corresponding parameter of the model (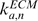) is a function of ECM density but not necessarily a linear one. Once formed, nascent adhesions will either turn over or mature to form stable adhesions. Like nascent adhesion formation, turnover is coupled to the rate of protrusion in the model, due to the transit of the nascent adhesions out of the lamellipodium [14],[21].

Nascent adhesions modulate Rac/PAK signaling in the model according to the mechanism outlined in the Introduction. Paxillin has many known binding and phosphorylation sites [22], several of which have been implicated in Rac activation; but in the current model we only consider Ser^273^, the site of GIT1/βPIX/PAK complex attachment. Nascent adhesions bound to phosphorylated paxillin thus mediate activation of Rac, which in turn activates PAK, and active PAK completes the feedback loop by phosphorylating paxillin.

The most speculative aspects of the model concern how stable adhesions impact protrusion, and we consider two effects: 1) stable adhesions enhance the recruitment and/or activity of myosin II, which generates mechanical tension and thus promotes further maturation (with a corresponding decrease in nascent adhesion density) [14],[16],[23]; and 2) stable adhesions directly antagonize protrusion, either by a mechanical or biochemical (e.g., Rho-mediated [17]) mechanism. The model parameters that determine the magnitudes of these two feedback loops (*E_s_* and *I_n_*, respectively) were varied systematically. Each effect can be turned off in the model by setting the value of its parameter to zero; i.e., the model encompasses the possibilities that either or both of these mechanisms is/are absent. Adhesions are considered immobile, and so as the leading edge protrudes, the effects of stable adhesions on other variables fade; stable adhesions also disassemble spontaneously.

### ECM density-dependent protrusion/adhesion phenotypes in CHO.K1 cells expressing paxillin-EGFP

**Figure 2:**
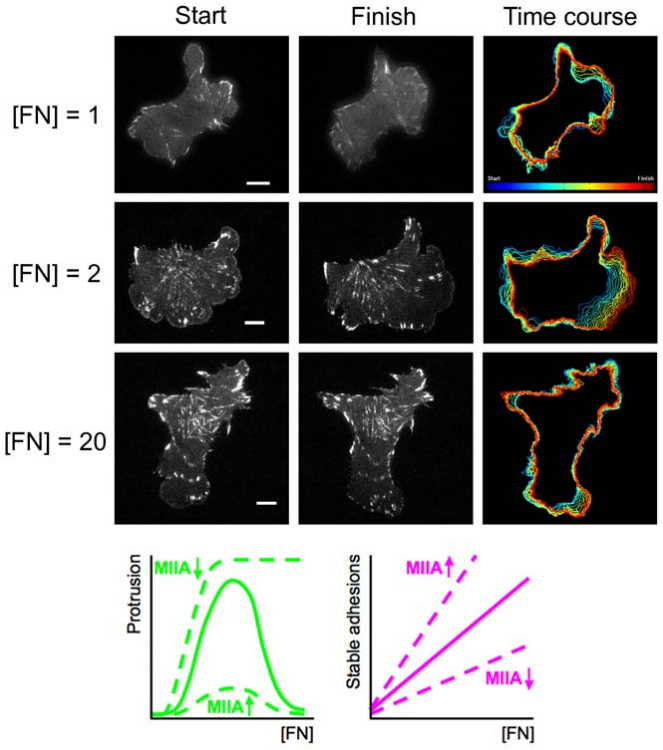
Qualitative dependence of CHO.K1 cell motility on ECM density. CHO.K1 cells expressing GFP-paxillin were filmed using TIRF microscopy as they migrated on the indicated densities of fibronectin ([FN], expressed as the coating concentration in µg/ml) for a period of 30 minutes. As the representative time courses show, only the intermediate concentration of 2 µg/ml supports a broad zone of persistent membrane protrusion. The images also show the monotonically positive dependence of stable adhesion abundance on [FN]. The illustrative plots show the predicted effects of myosin IIA (MIIA) depletion or overexpression.

To guide our analysis of the model, TIRF microscopy time courses of paxillin-EGFP-expressing CHO.K1 cells were acquired as they migrated on various fibronectin densities ([FN]); an intermediate [FN] (2 µg/ml coating concentration) is known to foster optimal cell migration speed of this cell line [9]. As seen in the representative cells shown in Fig. 2, one of the factors that contribute to this condition is an optimization of nascent adhesion abundance, seen as a diffuse enrichment of paxillin-EGFP at the leading edge, and of membrane protrusion motility relative to that which is supported at lower or higher [FN]. By inspection, protrusions under the suboptimal conditions are less persistent and restricted to smaller regions of the cell periphery, consistent with previous findings [17]. The other apparent trend observed in these experiments is a monotonic increase in the abundance of stable adhesions, seen as bright, oblate puncta of paxillin-EGFP, with increasing ECM density (Fig. 2).

Qualitative interpretation of these observations from the perspective of the model (Fig. 1) is straightforward. As the ECM density is increased, more total adhesions tend to be formed; however, very high ECM densities favor adhesion maturation rather than nascent adhesion turnover. Computational analysis of our quantitative model will show that this crossover from protrusive to adhesive phenotype is consistent with the opposing actions of feedback loops mediated by Rac and myosin.

### Exploration of the model parameter space by phase plane analysis reveals bistability in the hypothetical adhesion/protrusion circuit

The presence of feedback loops suggested that our model might be capable of bistability, a mathematical condition that is typically indicative of interesting dynamical behavior [24]. In this context, the manifestation of bistability is the capacity to produce, for the same values of the model parameters but depending on the initial conditions, both low and high protrusion states. Perhaps more relevant to the behavior of the stochastic model to follow, the region of bistability in parameter space separates regions that yield only low protrusion from those that yield only high protrusion at steady state.

**Figure 3:**
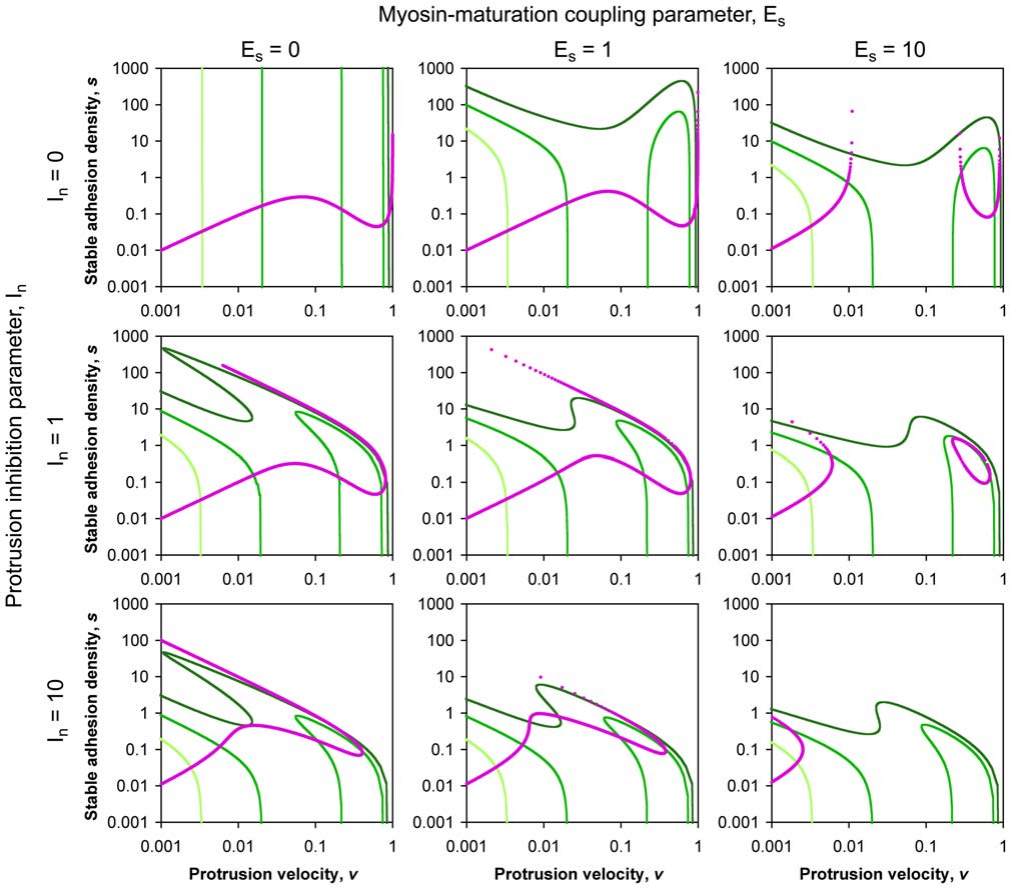
Exploration of model parameter space by phase plane analysis. The nullclines for *n* (green) and *s* (magenta) are plotted in (*v*, *s*) space. For the *n*-nullclines, the values of the ECM parameter are 0.03 (light green), 0.1 (green), and 0.3 (dark green) min^−1^. Intersections of the *n*- and *s*-nullclines are fixed points of the system. The values of *E_s_* and *I_n_* are varied as indicated, and all other parameters are assigned base case values (Supplementary Table S1).

The model's steady-state behavior was analyzed through classic phase plane analysis (for a biologically focused discussion, see [25]). The experimentally accessible protrusion velocity, *v* (expressed as a dimensionless fraction of its maximum value), is related mathematically to the densities of nascent and stable adhesions (*n* and *s*, respectively), allowing the *n*- and *s*-nullclines to be plotted in (*v*, *s*) space (Fig. 3). Nullclines demarcate adjacent regions in the phase plane where the variable in question increases and decreases in time, and therefore time-dependent trajectories in the phase plane tend to be attracted towards them, and the intersections of the *n*- and *s*-nullclines are fixed points of the system that are either stable (steady states) or unstable to perturbations.

**Figure 4:**
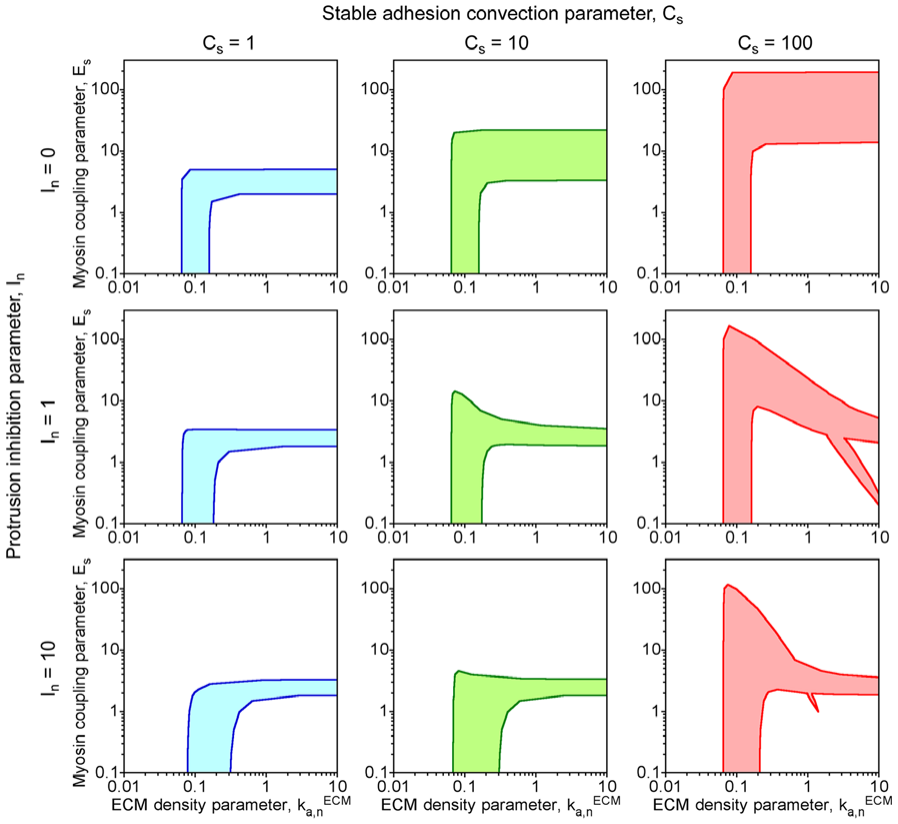
Regions of model bistability. In each plot, the shaded region of (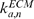, *E_s_*) parameter space indicates where there are multiple fixed points (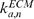 values given in units of min^−1^). Outside of these regions, the model is monostable, supporting either low (low 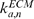 or high *E_s_*) or high (high 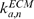 or low *E_s_*) protrusion. The values of *C_s_* and *I_n_* are varied as indicated, and all other parameters are assigned base case values (Supplementary Table S1).

Fig. 3 shows how the location(s) of the fixed point(s) are affected by the value of the ECM parameter (in each plot, the different *n*-nullclines) and those of the stable adhesion-mediated feedback parameters *E_s_* and *I_n_*. Analysis of many such phase plots with even finer changes in the parameter values allowed the construction of the graphs presented in Fig. 4, which delineate the regions of fixed-point multiplicity in (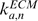, *E_s_*) parameter space for certain values of *I_n_* and the parameter describing convective transport of stable adhesions away from the leading edge, *C_s_* (hereafter, *C_s_* was fixed at a value of 10). Other model parameter values were also varied; however, it was found that only those that affect the coupling between stable adhesions and nascent adhesion-mediated signaling have a qualitatively distinctive effect on the conclusions. For example, changing the values of parameters characterizing the nascent adhesion-mediated positive feedback loop simply modulates up or down the range of 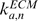 values for which bistability is observed (analysis not shown).

Taken together, these analyses establish the following conclusions. First, the hypothetical system is indeed capable of bistability, and the positive feedback inherent to the Rac/PAK signaling circuit is sufficient for this property. Even when the feedbacks from stable adhesions are turned off (*E_s_* = *I_n_* = 0), the system is bistable within a certain intermediate range of the ECM parameter (here, in the neighborhood of 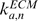
≈0.1 min^−1^; Fig. 4), whereas higher (lower) ECM densities promote only the high (low) protrusion state. From that point in parameter space, increasing the myosin coupling to adhesion maturation, characterized by the value of *E_s_* (moving up the vertical axis of each plot in Fig. 4), affects the bifurcation behavior of the system as follows. The system transitions through a region of parameter space where arbitrarily high ECM densities also support bistability and thus both high and low protrusion states, and finally into one in which only low protrusion is stable (adhesion maturation dominates). By comparison, increasing the value of the protrusion inhibition parameter, *I_n_*, is a more subtle perturbation; its primary outcome is “compression” of the *n*-nullclines down to lower stable adhesion density, since only at low *s* are appreciable values of *v* allowed (Fig. 3, with *I_n_* = 10). As we will show, this property has important consequences for the dynamical behavior of the system.

### Recapitulation of observed protrusion/adhesion phenotypes in stochastic simulations

Although phase plane analysis provides an instructive exploration of the parameter space and reveals the asymptotic behavior of the system in the limit of long times and large numbers of molecules, it cannot by itself recapitulate dynamic phenotypes. For this, we turned to stochastic simulations. This approach is deemed necessary when, as is the case here with stable adhesions, the numbers of certain model species in a particular region of the cell can be low or apparently zero much of the time. Indeed, in TIRF movies of paxillin-EGFP-expressing CHO.K1 cells, one observes the seemingly spontaneous emergence and stalling of leading edge protrusions, accompanied by the rapid assembly of a few nascent adhesions and a single adhesion maturation event, respectively [13],[14]. Furthermore, only a stochastic model will be able to produce what we envision to be two major phenomena governing leading edge dynamics: 1) stochastic “switching” between low and high protrusion states in the bistable regime, and 2) amplification of stochastic fluctuations to generate transient yet dramatic excursions from a particular stable state. In the case of the latter mechanism, there are two submodes: transient accelerations from an otherwise low protrusion state, and transient decelerations (pauses) from an otherwise persistent protrusion state.

Consistent with the phase plane analyses presented in Figs. 3 and 4, we systematically varied the values of the ECM parameter (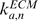) and those characterizing stable adhesion-mediated feedback (*E_s_* and *I_n_*), spanning the parameter space within and on either side of the bistable regime. Another key parameter that was varied here is N^*^, which scales the dimensionless variables of the model to discrete numbers of molecules found in a particular region at the cell periphery. The lower its value, the more likely the system will show stochastic, as opposed to deterministic, effects.

**Figure 5:**
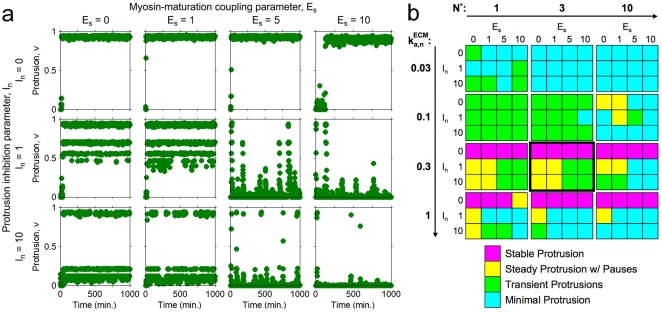
Characterization of protrusion/adhesion phenotypes through stochastic simulations. The model system was allowed to evolve stochastically, with all species numbers equal to zero initially. *a*. Protrusion velocity *v* is plotted as a function of time for 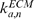 = 0.3 min^−1^, N^*^ = 3, and a matrix of *E_s_* and *I_n_* values as indicated. *b*. The same (*E_s_*, *I_n_*) matrix was repeated for different values of 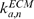 and N^*^ as indicated, and each simulation was coded according to the apparent phenotype. The matrix framed with a thicker border corresponds to the simulations shown in *a*. The raw data for each of these simulations, *v*(*t*) and *s*(*t*), are provided in Supplementary Figs. S1 and S2, respectively.

The results of these simulations, each initiated from all zero concentrations and spanning a period of 1,000 minutes, are summarized in Fig. 5 and presented more fully in Supplemental Figs. S1 and S2. We categorized each stochastic time course *v*(*t*) as exhibiting one of four characteristics: stable protrusion, steady protrusion with pauses, transient protrusions (defined as low protrusion punctuated by at least one brief excursion with *v* peaking at >0.5, not counting the initial transient), and minimal protrusion (low protrusion without any excursions as defined above). As expected, low ECM below the bistable transition generally supports only minimal protrusion. As the ECM parameter is increased, transient and then more stable protrusions emerge; consistent with experimental observations (Fig. 2), protrusion is inhibited at very high ECM density (provided that the values of *E_s_* and/or *I_n_* are sufficiently high), accompanied by a preponderance of stable adhesions. The transient protrusions emerge in the bistable region and also at intermediate ECM density just outside the bistable region. In the case of the latter, only low protrusion is stable, but the velocity can increase dramatically in tandem with stochastic loss of stable adhesions. A nonzero value of *I_n_* is important for this behavior, because it allows small numbers of stable adhesions to have a more dramatic effect, as observed experimentally. The scaling of the numbers of molecules in the system is also important; having more molecules gives rise to more deterministic behavior as anticipated, sometimes with stochastic switching events (Supplemental Figs. S1 and S2). An intermediate molecule number offers a compromise between overly noisy and purely deterministic behaviors.

### Diffusion of active Rac can propagate transient protrusion and adhesion maturation events across the leading edge

Although the standard stochastic simulation approach is insightful and at least qualitatively predictive with regard to experimental observations, there is an implicit assumption that the molecular components are either well-mixed or subject to processes that remain confined to a small region at the leading edge. Whereas the latter assumption is reasonable for species associated with immobile adhesion complexes, active Rac tends to be dispersed by lateral diffusion, and values of parameters associated with that process have been estimated from experimental data [26]. To account for Rac mobility, we performed spatially extended stochastic simulations using the Next Subvolume Method [27]. For the sake of simplicity and computational tractability, we considered diffusion in one spatial dimension only, corresponding in principle to the contour of the cell periphery.

**Figure 6:**
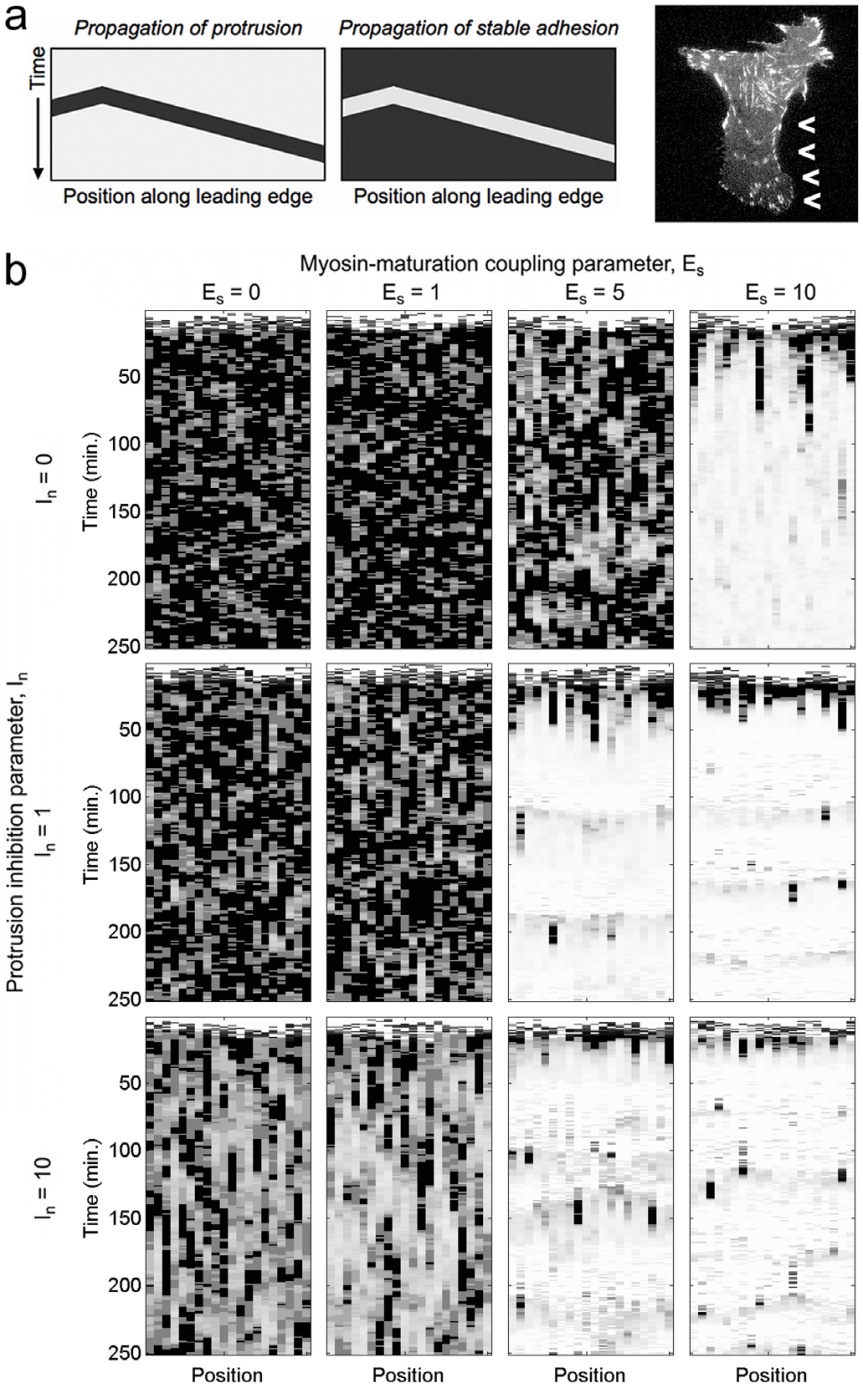
Alternating spatial waves of protrusion and adhesion. Spatially extended simulations were performed using the Next Subvolume Method, accounting for lateral diffusion of active Rac. Protrusion velocity is indicated in grayscale (white: *v* = 0; black: *v* = 1) as a function of time and position; the virtual leading edge is subdivided into 20 subvolumes, each 1.94 µm in length. Corresponding stable adhesion density maps are given in Supplementary Fig. S3. *a*. Propagating waves are perceived as contiguous regions radiating from the point of initiation. Evidence of membrane protrusion waves, each halted by a wave of adhesion maturation (arrowheads), is found in the cell from Fig. 2 plated on 20 µg/ml fibronectin. *b*. Velocity maps for a matrix of (*E_s_*, *I_n_*) values, parametrically consistent with the one-compartment simulations shown in Fig. 5 *a*.

The results, shown in Fig. 6 and Supplemental Fig. S3 for 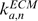 = 0.3 min^−1^, N^*^ = 3 (consistent with Fig. 5 *a*), are similar to those predicted by the standard, one-compartment simulations: high protrusion with varying degrees of pausing or low protrusion with varying degrees of transient bursting (one significant deviation was found in the case of *E_s_* = 10, *I_n_* = 0, which showed stable protrusion in the one-compartment case but not here in the spatially extended case; we attribute this to the tendency of diffusion to disperse and thus dilute the potency of active Rac in certain situations). The spatial simulations also show new phenomena, namely the tendency for transient protrusions (or transient pauses of protrusion) to propagate in a wave-like manner across all or at least a significant portion of the simulated leading edge; in CHO.K1 cells on high [FN], banded patterns of stable adhesions consistent with alternating waves of protrusion and adhesion maturation are observed (Fig. 6 *a*). In our simulations, such motility behavior is recovered with the higher values of *E_s_* and *I_n_*, i.e., when adhesion maturation antagonizes protrusion (Fig. 6 *b*); under these conditions, active Rac diffuses and triggers the protrusion feedback loop in adjacent locations, but the high protrusion state is not stable, and hence the protrusion wave dies out in concert with a band of adhesion maturation (Fig. S3).

In a somewhat more complicated model, we also considered that active PAK would have some propensity to exchange between immobilized, nascent adhesion-associated complexes and the highly mobile, cytosolic pool; however, at least for conditions where the spatial ranges of active Rac and PAK were similar, the results were not qualitatively different from those shown in Fig. 6 (results not shown).

### Experimental tests of the predicted relationship between myosin and adhesion maturation at different ECM densities

The phase plane analysis and stochastic simulations presented in the previous sections make a specific prediction about the role of myosin-mediated adhesion maturation in shaping the dependence of membrane protrusion dynamics on ECM density. The model supposes that stable adhesions antagonize protrusion, and relief of that inhibition by reducing myosin levels (conceptualized in the model as lowering the value of *E_s_*) is predicted to have more dramatic effects at higher ECM densities. At low ECM density, the interpretation of the model is that membrane protrusion is not constrained by mature adhesions or myosin II but rather by low efficiency of nascent adhesion formation and therefore insufficient Rac/PAK signaling.

**Figure 7:**
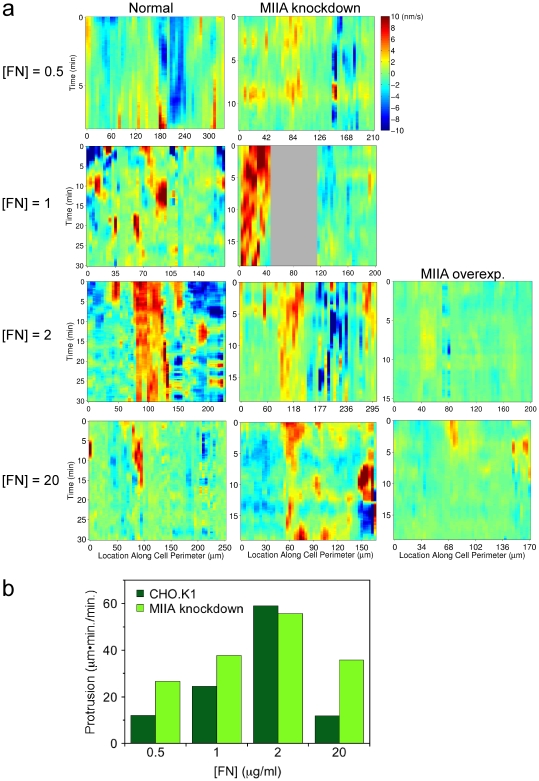
Effects of myosin IIA knockdown or overexpression assessed by protrusion velocity mapping. CHO.K1 cells expressing fluorescently labeled paxillin were filmed using TIRF microscopy as they migrated on the indicated densities of fibronectin ([FN], expressed as the coating concentration in µg/ml). Myosin IIA levels were knocked down by RNA interference or increased by overexpression as indicated. *a*. For each condition, the velocity of protrusion is mapped for a representative cell as a function of time and position around the cell periphery. A gray area indicates a region of the cell periphery where protrusion velocity could not be determined. *b*. A protrusion event is marked as a contiguous region in space and time with protrusion velocity exceeding 1 nm/s and containing at least one instance where the protrusion velocity exceeded 5 nm/s (Supplemental Fig. S4). The total area of such protrusions on the velocity map (in units of length*time), normalized by the duration of the experiment, is plotted for normal and myosin IIA knockdown conditions from *a*.

To test this prediction, myosin IIA was either knocked down by RNA interference or overexpressed in CHO.K1 cells expressing fluorescent paxillin, and these cells were filmed as they migrated on various [FN] as in Fig. 2. For comparison, the protrusion velocity around the periphery of each cell was mapped as a function of time (Fig. 7 *a*), and the total protrusive activity was quantified (Fig. 7 *b*; see the figure caption and Supplemental Fig. S4 for details). Consistent with the model, myosin IIA knockdown enhances protrusion, and the effect relative to the normal myosin IIA level/activity is to broaden the range of [FN] over which sustained protrusion is supported. Thus, whereas protrusion is normally restrained at the highest [FN] (20 µg/ml), in myosin IIA-depleted cells the protrusion pattern at high [FN] is more comparable to optimal protrusion at intermediate [FN]. As expected, this effect of myosin IIA knockdown is noticeably reduced as the [FN] is progressively reduced, with only transient protrusions at the lowest [FN] tested (0.5 µg/ml). Myosin IIA overexpression abrogated protrusion and increased stable adhesion abundance as expected (Fig. 7 *a*).

## Discussion

Computational modeling has emerged as a useful tool for integrating the multiple, competing subprocesses that govern cell motility and migration, an approach that has advanced the field by generating non-intuitive and in some cases quantitative insights and predictions [28]. The model offered here incorporates molecular mechanisms of integrin-mediated Rac/PAK signaling, which underlies a known positive feedback loop that promotes protrusion of lamellipodia. The excitability of the Rac/PAK feedback loop and its regulation by processes that enhance adhesion maturation readily account for observed motility phenotypes and their dependence on ECM density. Systems with opposing feedback loops tend to be prone to bistability, such that certain sets of parameter values foster both low and high activity states; our model is no exception, but the results suggest that this property is not necessary for generating transient protrusion and adhesion maturation phenomena. Rather, the relevance of this region of parameter space is that it straddles those regions that give monostable low and monostable high protrusion. In the vicinity of that interface, the stochastic model readily produces transient but potentially dramatic excursions from the stable state, be they transient accelerations or decelerations of protrusion.

Not surprisingly, what does appear to be critical for the stochastic behavior of the model is the overall abundance of adhesions. If the density of nascent adhesions required to ignite positive feedback is too high, one will tend to achieve deterministic behavior; this suggests that the feedback circuit is endowed with *absolute* sensitivity, responding to the appearance of a reasonably low density of nascent adhesions, in addition to *relative* sensitivity to small gains in nascent adhesion density. Likewise, the regulatory influence of myosin must also possess absolute sensitivity to the presence of stable adhesions. This is considered quite plausible, as CHO.K1 cells typically exhibit qualitative changes in leading edge dynamics coinciding with the initial emergence of nascent/stable adhesions in a region that was theretofore adherent/protrusive.

Two issues that must be confronted in any mathematical modeling effort are completeness and generality. Especially for models of cellular processes, one must be judicious about the level of detail to include. The coarseness of a model should generally be determined by how well the constituent mechanisms are understood and the need to specify values of their corresponding rate parameters, always with an eye towards the research questions being asked [29]. The focus of the model presented here is limited to leading edge dynamics and is not meant to predict the propensity for overall cell translocation; other published models have addressed the mechanics of overall cell migration, with correspondingly less attention paid to the biochemical signaling aspects [30]–[32]. Furthermore, the model contains semi-mechanistic and phenomenological elements that should be refined once new data, especially those of a quantitative nature, come to light. In particular, there is a void in our understanding of the feedback loops mediated by stable adhesions, which more than any other features of the model dictate its qualitative behavior. Although the role of myosin II in promoting adhesion maturation is well established, the precise physicochemical mechanism awaits further characterization, as does the mechanism(s) by which stable adhesions might hinder leading edge protrusion as postulated here. The latter might also depend on myosin II activity, for example through a contribution to retrograde actin flow. Other missing details that might inform refinement of the model include those related to nascent adhesion formation and the nature of the coupling between actin polymerization and the lifetime of nascent adhesions [20]. Another axis along which the model should be updated is the inclusion of other integrin-mediated signaling pathways, most notably the roles of focal adhesion kinase, Src, and RhoA [33],[34].

As noted above, another issue to bear in mind is the generality of the conclusions across different contexts. Our analysis was guided by observations of CHO.K1 cells on fibronectin-coated glass, a well characterized system; however, each cell line/type is expected to have its particular nuances with regard to trade-offs among protrusion, adhesion, and contractility [19]. Hence, the central question is whether or not similar feedback structures give rise to spatiotemporal waves of protrusion and/or adhesion in other cell types [35],[36]. Our speculation is that they do in cells that are more or less mesenchymal in origin, perhaps with different parameter “tunings”, whereas leukocytes and other cells that move via amoeboid motility might be regulated by fundamentally distinct mechanisms. The concept of variations in the model parameters might be relevant for explaining heterogeneity within a cell population and even differences between distant regions of the same cell that define what is and what is not a leading edge. Indeed, in CHO.K1 cells the segregated recruitment and activation of myosins IIA and IIB at the cell front and rear, respectively, is one mechanism by which contractility-dependent processes in particular are polarized [37].

## Materials and Methods

### Cell culture and TIRF microscopy

CHO.K1 cells were cultured under standard conditions and transfected with paxillin-EGFP [38] using Lipofectamine (Invitrogen). Where applicable, myosin IIA expression was knocked down by RNA interference by co-transfection with the pSUPER-IIA plasmid, used in 10∶1 excess to paxillin-EGFP to ensure knockdown in cells emitting fluorescence, as described previously [16]. Myosin IIA overexpression was achieved by expression of EGFP-myosin IIA, co-transfected with paxillin fused to CoralHue monomeric Kusabira Orange (mKO) in place of EGFP [14].

Transfected cells were plated on fibronectin-coated glass-bottomed dishes in CCM1 (HyClone) for 1 hour and maintained at 37°C at pH 7.4. TIRF images were acquired using an Olympus IX70 inverted microscope fitted with a 1.45 NA PlanApo 60× TIRFM objective and modular automation controller (Ludl Electronic Products), controlled by Metamorph software (Molecular Devices). EGFP was excited using the 488 nm laser line of an Ar ion laser (Melles Griot), and a Z488RDC dichroic mirror (Chroma Technologies) was used. For dual EGFP/mKO acquisition, a polychroic mirror (Z488/543rpc), dual band-pass filter (Z488/543) and HQ525/50 and HQ620/60 emission filters (all from Chroma Technologies) were used. Images were acquired with the Retiga Exi charge-coupled device camera (Qimaging).

### General modeling considerations

Certain parameters of the model are dimensionless and phenomenological; these are classified by whether they characterize enhancement of species *i* formation (*E_i_*), inhibition of species *i* formation (*I_i_*), or augmentation of species *i* consumption rate (*C_i_*). Other parameters have dimensions and include first-order rate constants with units of inverse time, characterizing assembly/activation or disassembly/deactivation of species *i* (*k_a,i_* or *k_d,i_*, respectively), and diffusion coefficients with units of area/time (*D_i_*). Dimensionless parameters *K_i_* denote ratios of rate constants, characterizing the rate of assembly or activation relative to that of disassembly or deactivation for species *i* (*K_i_* = *k_a,i_*/*k_d,i_*). Values of all model parameters are given in Table S1 of the Supplemental Material; for those parameters that were varied, the values are specified in the corresponding Figure captions.

### Deterministic model equations

Based on the assumptions laid out in the main text and illustrated in Fig. 1, the dimensionless densities of nascent adhesions (*n*), stable adhesions (*s*), and recruited myosin (*m*), are conserved as follows.
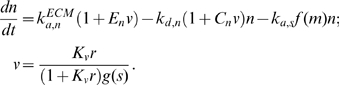
(1)

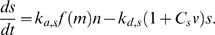
(2)

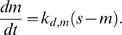
(3)The value of the parameter 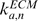 maps in some way to the density and character of the ECM, and *v* is the dimensionless protrusion velocity. The phenomenological functions *f*(*m*) and *g*(*s*) characterize the effects of myosin and stable adhesions on adhesion maturation and protrusion inhibition, respectively. Various forms of these functions might be imposed; for example, for a “force-based” dependence [39], one might be inclined to use 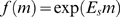 and/or 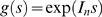. Because the underlying mechanisms are as yet unclear, we currently prefer to use the more conservative linear functions,
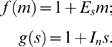
(4)The equations for the signaling circuit variables are as follows. The variable *x* represents the subset of *n* harboring phosphorylated paxillin (and, implicitly, GIT1/βPIX/PAK complexes), *r* is the density of active Rac (activated by βPIX), and *p* is the subset of *x* harboring activated PAK (activated by Rac).
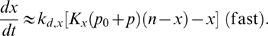
(5)

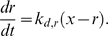
(6)

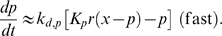
(7)The small basal paxillin phosphorylation activity, *p*_0_, is included in Eq. 5 so that *x*, *r*, and *p* can evolve in time when all initial values are zero.

### Phase plane analysis

The *n*- and *s*-nullclines are defined as the conditions for which *dn*/*dt* = 0 (Eq. 1) and *ds*/*dt* = 0 (Eq. 2), respectively, assuming all other (faster responding) variables to be quasi-steady. Therefore, one takes *m* = *s* (Eq. 3), and *n* is related to *v* by the steady-state solution of Eqs. 5–7. Parameters that were not varied as indicated were assigned base case values (Table S1, Supplemental Material).

### Stochastic, one-compartment simulations

Stochastic simulations were carried out using the standard First Reaction Method [40], implemented in MATLAB (MathWorks, Natick, MA; we have since implemented the model using the Next Reaction Method [41]). Dimensionless variables (lowercase) in Eqs. 1–7 are converted to numbers of molecules (uppercase) by specifying a number density scaling factor for each variable, denoted in uppercase with an asterisk superscript; for example *n* = *N*/*N* ^*^. Based on the formulation of Eqs. 1–7, 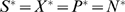, and from the definition of *K_i_*, 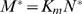 and 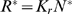. Therefore, the additional parameters *N* ^*^, *K_m_*, and *K_r_* must be specified for stochastic simulations. Whereas *N* ^*^ was varied, we generally assumed *K_m_* = *K_r_* = 10; i.e., the numbers of *R* and *M* are somewhat amplified relative to their activators (*X* and *S*, respectively). Under the conditions explored here, the values of these parameters do not significantly affect the model output. Additional details related to the implementation of stochastic simulations are provided in Supplemental Text S1.

### Stochastic, spatial simulations using the Next Subvolume Method

These simulations were implemented in MATLAB, following the algorithm described in detail by Elf et al. [27]. In all cases, diffusion of active Rac is in one spatial dimension with periodic boundary conditions, with a diffusion coefficient value of *D_r_* = 15 µm^2^/min (0.25 µm^2^/s) as estimated by Moissoglu et al. [26]. The lifetime of active Rac on the membrane was estimated in the same study, from which we obtained *k_d,r_* = 4.0 min^−1^; this sets the characteristic length scale for Rac diffusion, *L_r_* = (*D_r_*/*k_d,r_*)^1/2^≈2 µm, which we used as the node spacing. Thus, the frequency of an active Rac molecule hopping from one spatial node to each of the two adjacent nodes is set to *D_r_*/*L_r_* ^2^ = *k_d,r_* = 4.0 min^−1^. Additional details related to the implementation of stochastic simulations, including consideration of Rac diffusion in two dimensions, are provided in Supplemental Text S1.

### Construction of velocity maps from image data

Cell images were converted to binary structures based on manual thresholding of the fluorescent paxillin intensity, and cell outlines were smoothed using sequential dilation and erosion operations (MATLAB image analysis toolbox). At equidistant points along the cell perimeter, cell protrusion velocity was estimated as the change in cell edge location per unit time along lines placed normal to the boundary of the cell in the first image. A moving average over 20 frames (100 seconds) was used to smooth out temporal fluctuations.

## Supporting Information

Table S1 Model parameters.
(0.09 MB PDF)

Text S1 Stochastic modeling details.
(0.72 MB PDF)

Figure S1Raw simulation results corresponding to the analysis shown in Fig. 5 b. Protrusion velocity v is plotted as a function of time for the indicated values of 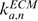 and N^*^ and a matrix of *E_s_* and *I_n_* values as indicated in Fig. 5 a.(0.38 MB PDF)

Figure S2Raw simulation results corresponding to the analysis shown in Fig. 5 b. Stable adhesion number S is plotted as a function of time for the indicated values of 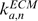 and N^*^ and a matrix of *E_s_* and *I_n_* values as indicated in Fig. 5 a.(0.63 MB PDF)

Figure S3 Spatially extended simulations were performed using the Next Subvolume Method, accounting for lateral diffusion of active Rac. Stable adhesion number S is indicated by the color scale as shown (red: S = maximum; black: S = 0) as a function of time and position. These results are from the same simulations used to generate the protrusion velocity results shown in Fig. 6.
(0.30 MB PDF)

Figure S4 The velocity maps in Fig. 7 are shown in segmented form here. Red regions: velocity (nm/s) >5.0; yellow regions: 1.0< velocity (nm/s) <5.0 and contacting a red region; white regions: 1.0< velocity (nm/s) <5.0 but not contacting a red region; black regions: velocity (nm/s) <1.0. The contiguous red/yellow regions are considered bona fide protrusions.
(0.28 MB PDF)
