# Supplementary material for: Stochastic Model of Integrin-Mediated Signaling and Adhesion Dynamics at the Leading Edges of Migrating Cells
Source: PLoS Comput Biol. 2010 Feb 26;6(2):e1000688. doi: 10.1371/journal.pcbi.1000688 (PMC2829041; doi:10.1371/journal.pcbi.1000688)
Supplement: Table S1 — Model parameters. (0.09 MB PDF) [file pcbi.1000688.s001.pdf]

Parameter	*Description*	*Min value*	*Base value*	*Max value*	*Comment*
$k_{a,n}^{ECM}$ (min^{-1})	Rate constant, N assembly	0.01		10	Varied; input to the model
E_n	Rac → protrusion coupling		100		Arbitrarily >> 1 (strong effect)
K_v	Saturation of protrusion velocity		1		Max protrusion requires r >> 1
I_n	S → protrusion inhibition	0		10	Varied over a broad range
$k_{d,n}$ (min^{-1})	Rate constant, basal N turnover		0.1		Arbitrary; same value as $k_{d,s}$
C_n	Protrusion → N turnover coupling		20		Max turnover $C_n k_{d,n} \approx 2$ min^{-1}; ref. [1]
$k_{a,s}$ (min^{-1})	Rate constant, basal S growth		0.01		Arbitrarily << $k_{d,s}$
E_s	Myosin → S growth coupling	0		100	Varied over a broad range
$k_{d,s}$ (min^{-1})	Rate constant, S disassembly		0.1		Time scale ~ 10 min.; ref. [1]
C_s	Protrusion → S convection	1	10	100	Same order of magnitude as C_n
$k_{d,m}$ (min^{-1})	Rate constant, M deactivation		4		Arbitrarily fast (same as $k_{d,r}$)
$k_{d,x}$ (min^{-1})	Rate constant, X dephosphorylation		10		Arbitrarily fast
K_x	Saturation of phospho-paxillin		1		Not saturated when p ~ 1
p_0	Basal paxillin phosphorylation		0.01		Arbitrarily << 1
$k_{d,r}$ (min^{-1})	Rate constant, Rac deactivation		4		Fixed from ref. [2]
$k_{d,p}$ (min^{-1})	Rate constant, PAK deactivation		10		Arbitrarily fast
K_p	Saturation of PAK activation		1		Not saturated when r ~ 1
N^*	Scaling factor, N	1	3	10	Stochastic models; varied
K_m	Amplification factor, S → Myosin		10		Stochastic models; see main text
K_r	Amplification factor, Paxillin → Rac		10		Stochastic models; see main text
D_r (μm^2/min)	Mobility coefficient, Rac		15		Next subvolume model; fixed from ref. [2]

Table S1. Model parameters.

References:
1. Nayal A, Webb DJ, Brown CM, Schaefer EM, Vicente-Manzanares M, et al. (2006) Paxillin phosphorylation at Ser273 localizes a GIT1-PIX-PAK complex and regulates adhesion and protrusion dynamics. J Cell Biol 173: 587-599.
2. Moissoglu K, Slepchenko BM, Meller N, Horwitz AF, Schwartz MA (2006) In vivo dynamics of Rac-membrane interactions. Mol Biol Cell 17: 2770-2779.
