# Supplementary material for: Stochastic Model of Integrin-Mediated Signaling and Adhesion Dynamics at the Leading Edges of Migrating Cells
Source: PLoS Comput Biol. 2010 Feb 26;6(2):e1000688. doi: 10.1371/journal.pcbi.1000688 (PMC2829041; doi:10.1371/journal.pcbi.1000688)
Supplement: Text S1 — Stochastic modeling details. (0.72 MB PDF) [file pcbi.1000688.s002.pdf]

Supplemental Material

"Stochastic Model of Integrin-mediated Signaling and Adhesion Dynamics at the Leading Edges of Migrating Cells," by Cirit et al.

Text S1: Stochastic Modeling Details

One-compartment simulations

Here we define the propensity functions for each of the stochastic transitions in the model. As prescribed in the main text, we relate dimensionless model variables to numbers of molecules by scaling factors, indicated with an asterisk, e.g. $N = N^* n$, where N is the absolute number of nascent adhesions in the control volume and n is the corresponding dimensionless variable. Based on the formulation of Eqs. 1-7 for the dimensionless variables, the other scaling factors are related to N^* as follows.

$$S^* = X^* = P^* = N^*$$
$$M^* = K_m N^*$$
$$R^* = K_r N^*$$

The present model contains both mechanistic and phenomenological elements, and therefore the stochastic version of the model is not automatically specified in the way that a mass-action model would be. Our propensity functions, akin to reaction rates (given in units of number of molecules per minute), are as follows.

Nascent adhesion assembly ($\varnothing \rightarrow N$):	$k_{a,n}^{ECM}\left(1 + E_n v\right)N^*$
Nascent adhesion turnover ($N \rightarrow \varnothing$):	$k_{d,n}\left(1 + C_n v\right)N$
Adhesion maturation ($N \rightarrow S$):	$k_{a,s} f(m) N$
Disappearance of stable adhesions ($S \rightarrow \varnothing$):	$k_{d,s}\left(1 + C_s v\right)S$
Myosin activation ($\varnothing \rightarrow M$):	$k_{d,m} K_m S$
Myosin deactivation ($M \rightarrow \varnothing$):	$k_{d,m} M$
Paxillin phosphorylation ($\varnothing \rightarrow X$):	$k_{d,x} K_x \left(p_0 + p\right)\left(N - X\right)$
Paxillin dephosphorylation ($X \rightarrow \varnothing$):	$k_{d,x} X$
Rac activation ($\varnothing \rightarrow R$):	$k_{d,r} K_r X$
Rac deactivation ($R \rightarrow \varnothing$):	$k_{d,r} R$

PAK activation ($\varnothing \rightarrow P$): $k_{d,p}K_p r(X - P)$

PAK deactivation ($P \rightarrow \varnothing$): $k_{d,p}P$

In these functions, the dimensionless values r, s (v is a function of r and s), m, and p are calculated according to $r = R/R^*$, etc.

One point that warrants additional discussion is the way in which paxillin phosphorylation and PAK activation are handled above. As in the deterministic model, we collectively account for turnover and maturation of all nascent adhesions N; N subsumes X (nascent adhesion complexes with phosphorylated paxillin), which in turn subsumes P (nascent adhesion complexes harboring active PAK). An alternative way to model this is to define N as the subset of nascent adhesions without paxillin phosphorylated; handled that way, the propensity functions for $\varnothing \rightarrow X$ and $X \rightarrow \varnothing$ above would be replaced by $N \rightarrow X$ and $X \rightarrow N$, respectively (with $N - X$ replaced with N), and additional transitions $X \rightarrow \varnothing$ (turnover) and $X \rightarrow S$ (maturation) would be needed. Accordingly, one would be inclined to define X as the subset of nascent adhesions with paxillin phosphorylated but not harboring active PAK, in which case the propensity functions for $\varnothing \rightarrow P$ and $P \rightarrow \varnothing$ above would be replaced by $X \rightarrow P$ and $P \rightarrow X$, respectively (with $X - P$ replaced with X), and additional transitions $P \rightarrow \varnothing$ (turnover) and $P \rightarrow S$ (maturation) would be needed; in the propensity function for $\varnothing \rightarrow R$, one would replace X with $X + P$. These two model variations give approximately identical results provided that the values of the rate constants $k_{d,x}$ and $k_{d,p}$ are sufficiently large (consistent with the assumption of fast kinetics in Eqs. 5 and 7). Rapid equilibration among N, X, and P also ensures that the quantities $N - X$ and $X - P$ remain ≥ 0 throughout the simulation.

Spatially extended simulations

As explained in the main text, we implemented spatially extended stochastic simulations using the Next Subvolume Method. In addition to the reaction propensities specified in the previous section, the spatial simulations account for diffusion of species i between adjacent compartments, each modeled as a "hopping" reaction with first-order rate constant D_i/L^2, where D_i is the diffusivity of species i, and L is the node spacing between adjacent compartments. This formulation may be applied to systems with $d = 1, 2$, or 3 spatial dimensions, where each compartment has $2d$ nearest neighbors. Thus, in the absence of other reactions, the mean-squared displacement of species i is equal to $2dD_it$, where t is time.

A suitable compromise between numerical accuracy and computational expense is generally achieved by setting the node spacing L equal to the smallest of the dynamic length scales, $L_i = \sqrt{D_i \tau_i}$, where τ_i is the mean lifetime of diffusible species i. In the simulations shown in Fig. 6, only active Rac (r) is diffusible, and $\tau_r = 1/k_{d,r}$. As explained in the main text, we used literature estimates of D_r and $k_{d,r}$ to obtain $L = L_r \approx 2$ μm.

We assume a one-dimensional ($d = 1$) geometry, corresponding to the contour of a leading edge, with periodic boundary conditions. To be certain, this is an abstraction of the actual system (see the illustration below). As the leading edge advances, adhesions move relative to the frame of reference, in the dimension orthogonal to the leading edge, by convection. This is handled implicitly in the model. For reasons that are not yet completely clear, the zone where nascent adhesions are found is apparently confined to that of actin polymerization/depolymerization at the leading edge; accordingly, nascent adhesion turnover depends on dimensionless protrusion velocity v because this determines how fast nascent adhesions arrive at the rear of this zone. The disappearance of stable adhesions also depends on v but for a different reason: it is reasoned that the influence of stable adhesions on protrusion is progressively reduced as the leading edge moves farther away from them.

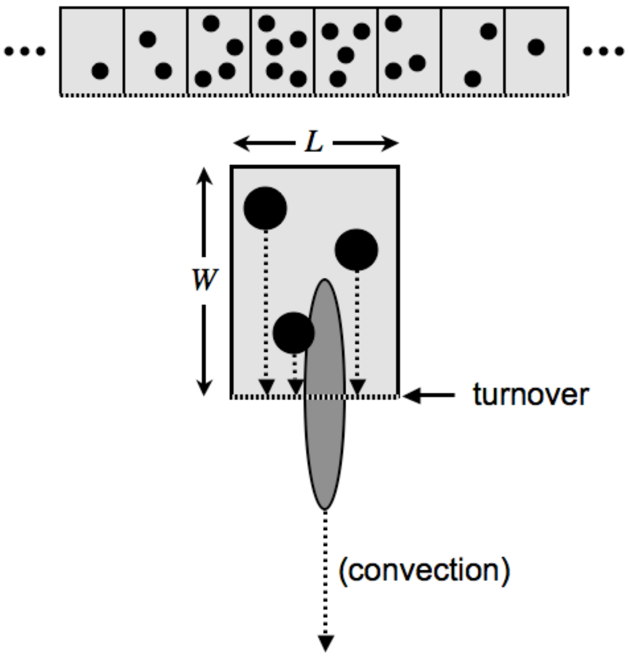

Rac diffuses laterally in the plane of the membrane, i.e. both along and orthogonal to the leading edge. What is not explicitly accounted for in the model is the loss of active Rac by net diffusion from the rear of the modeled domain. If we assign a dimension W to represent the width of each compartment in the direction orthogonal to the leading edge (corresponding to the thickness of the nascent adhesion zone described above), the critical question is the size of W relative to L_r. It is readily estimated that the fraction of active Rac molecules that would be lost to the interior of the cell is given by

$$\frac{L_r}{W}\frac{\tanh(W/L_r)}{1+\tanh(W/L_r)},$$

the remaining fraction being lost to deactivation within the modeled domain. Perhaps not coincidentally, indications are that $W \sim L_r$. For $W = L_r$, 43% of the active Rac molecules generated are lost by net diffusion; in that case one might be inclined to adjust the value of $k_{d,r}$ by a factor of $(1 - 0.43)^{-1} = 1.76$. Alternatively, considering that the adhesion dynamics are considerably slower than the time scale of $1/k_{d,r}$, one could simply reduce the definition of the node spacing according to

$$L = L_r\left(1-\frac{L_r}{W}\frac{\tanh(W/L_r)}{1+\tanh(W/L_r)}\right)^{1/2},$$

i.e., $L = 0.75L_r$ for $W = L_r$. Such an adjustment was deemed unnecessary in our analysis, because its magnitude is probably not larger than the uncertainties associated with the values of D_r, $k_{d,r}$, and W.
