# Supplementary material for: Stochastic Model of Integrin-Mediated Signaling and Adhesion Dynamics at the Leading Edges of Migrating Cells
Source: PLoS Comput Biol. 2010 Feb 26;6(2):e1000688. doi: 10.1371/journal.pcbi.1000688 (PMC2829041; doi:10.1371/journal.pcbi.1000688)

Fig. S1 A. Molecule number scaling: $N^* = 1$

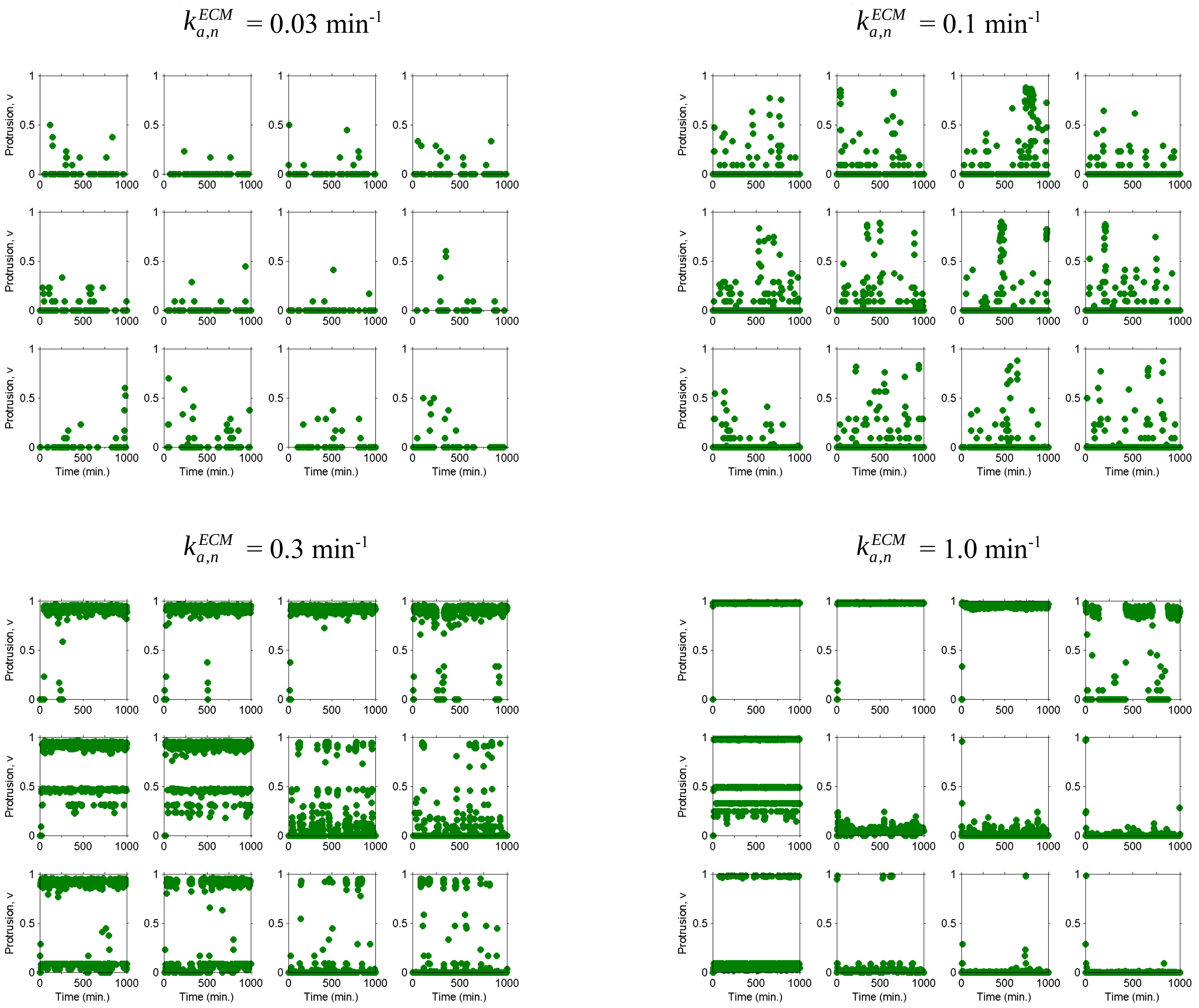

Fig. S1 B. Molecule number scaling: $N^* = 3$

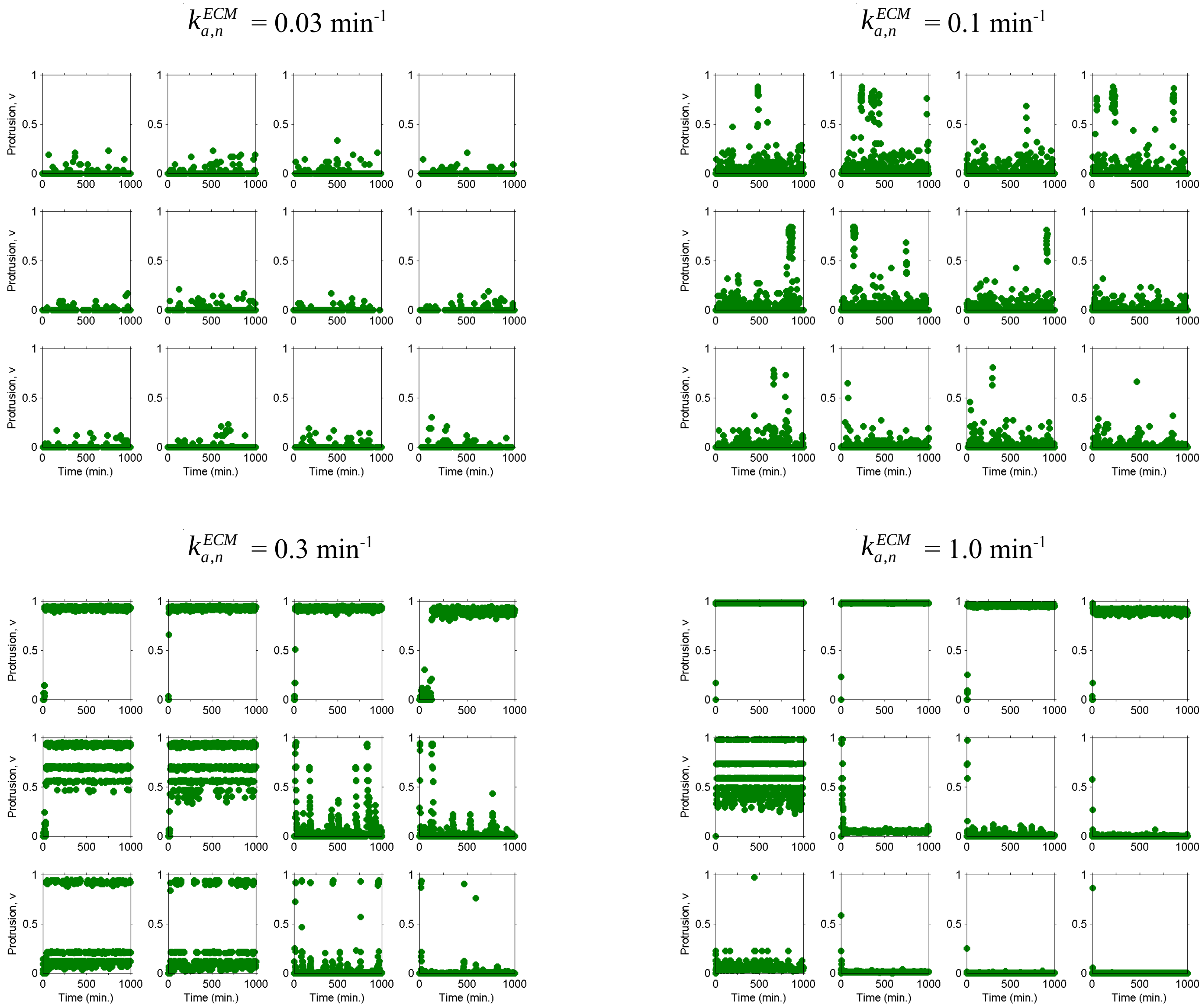

Fig. S1 C. Molecule number scaling: $N^{*} = 10$

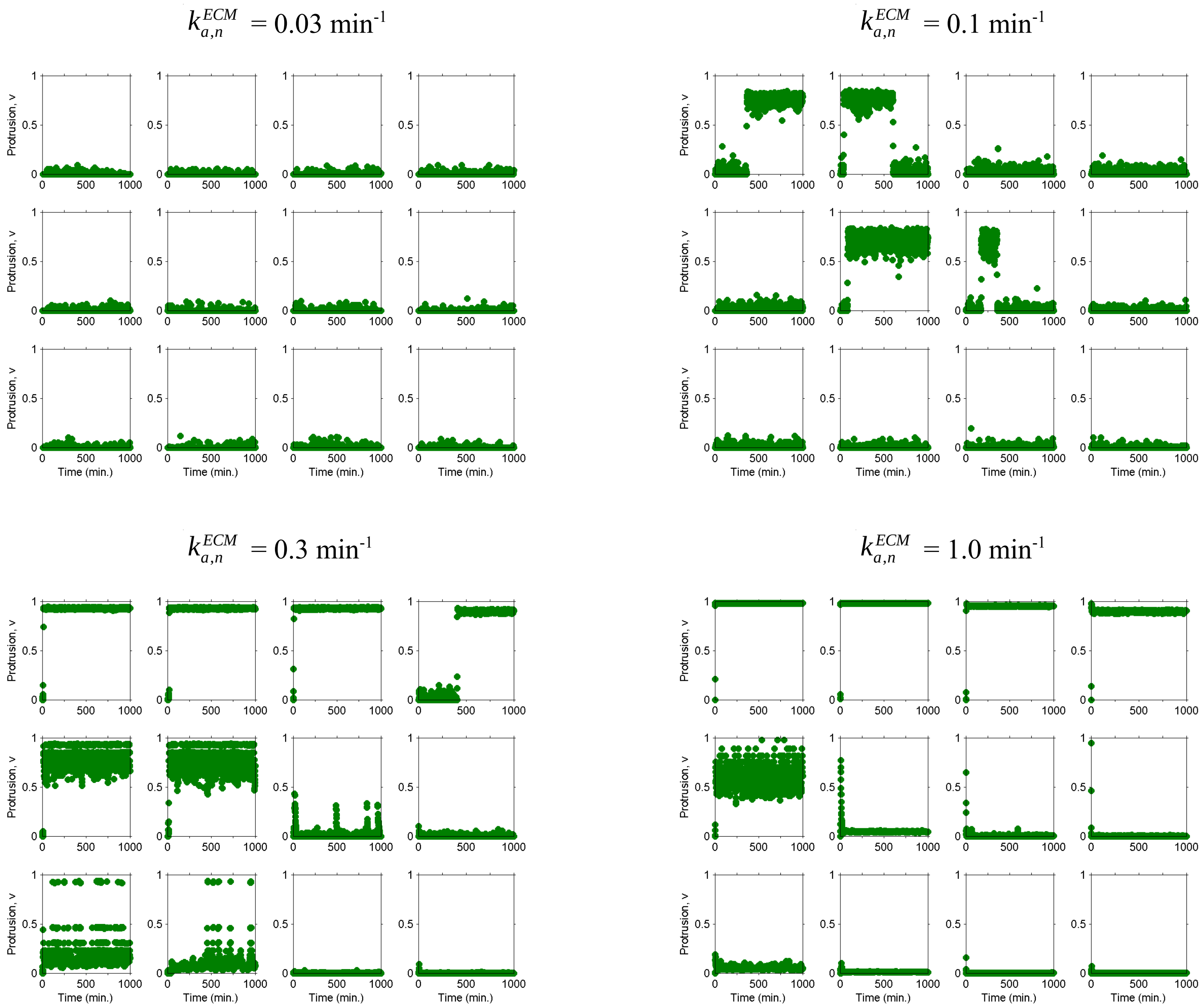

Supplement: Figure S1 — Raw simulation results corresponding to the analysis shown in Fig. 5 b. Protrusion velocity v is plotted as a function of time for the indicated values of and N* and a matrix of Es and In values as indicated in Fig. 5 a. (0.38 MB PDF) [file pcbi.1000688.s003.pdf]
