# Supplementary material for: Stochastic Model of Integrin-Mediated Signaling and Adhesion Dynamics at the Leading Edges of Migrating Cells
Source: PLoS Comput Biol. 2010 Feb 26;6(2):e1000688. doi: 10.1371/journal.pcbi.1000688 (PMC2829041; doi:10.1371/journal.pcbi.1000688)

Fig. S2 A. Molecule number scaling: $N^* = 1$

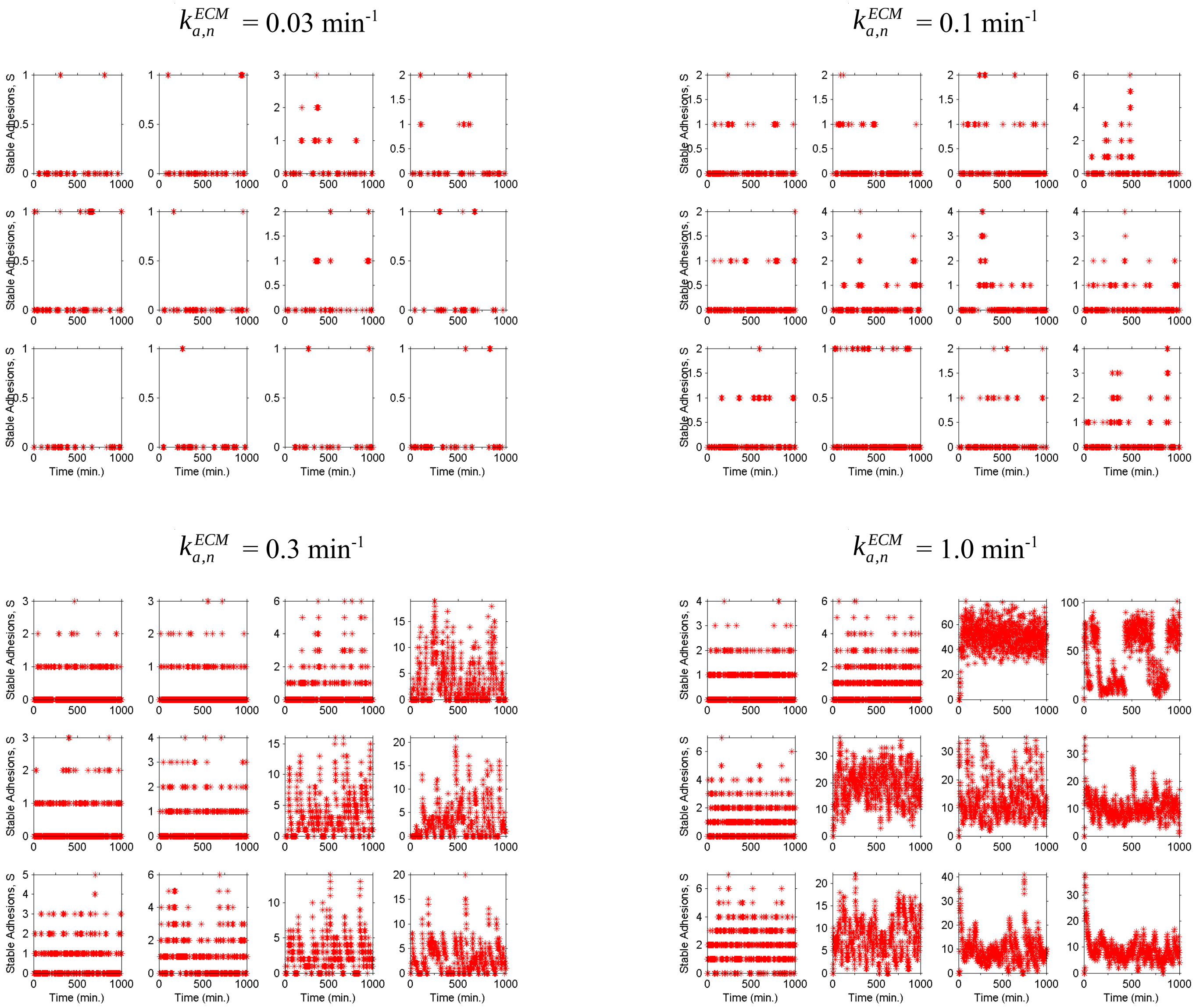

Fig. S2 B. Molecule number scaling: $N^{*} = 3$

$k_{a,n}^{ECM} = 0.03\ \text{min}^{-1}$

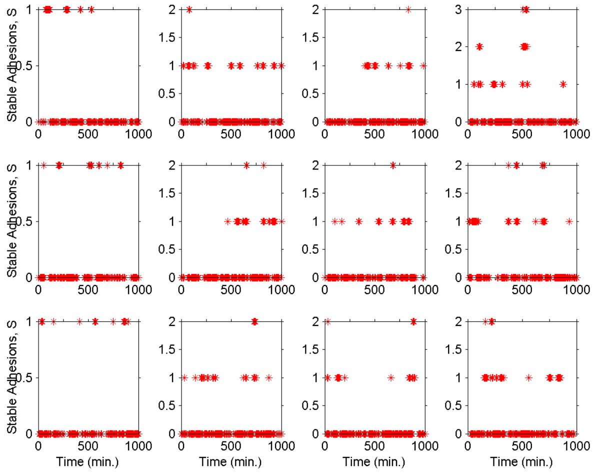

$k_{a,n}^{ECM} = 0.1\ \text{min}^{-1}$

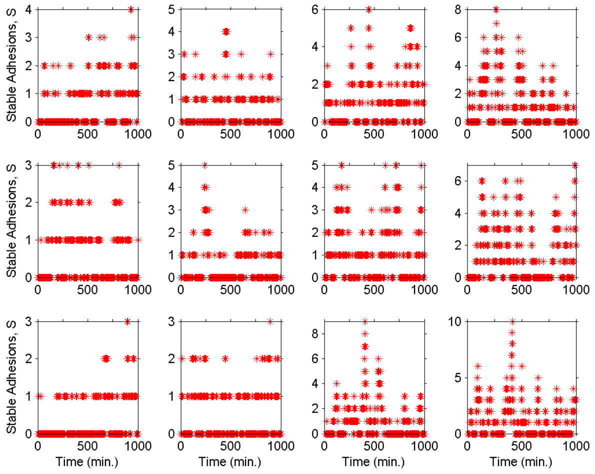

$k_{a,n}^{ECM} = 0.3\ \text{min}^{-1}$

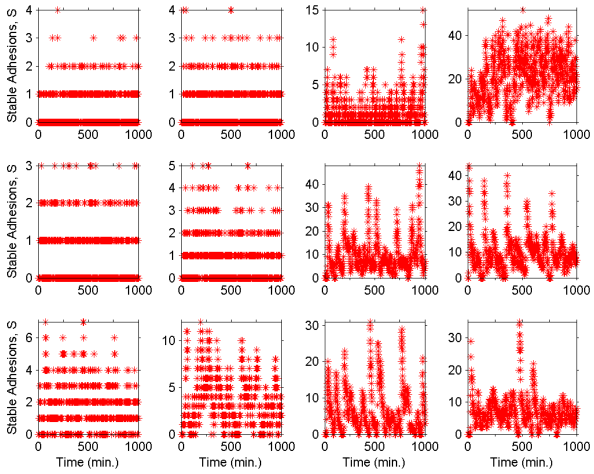

$k_{a,n}^{ECM} = 1.0\ \text{min}^{-1}$

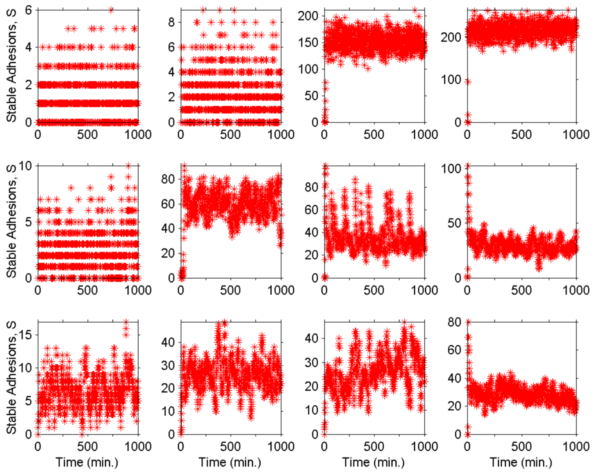

Fig. S2 C. Molecule number scaling: $N^{*} = 10$

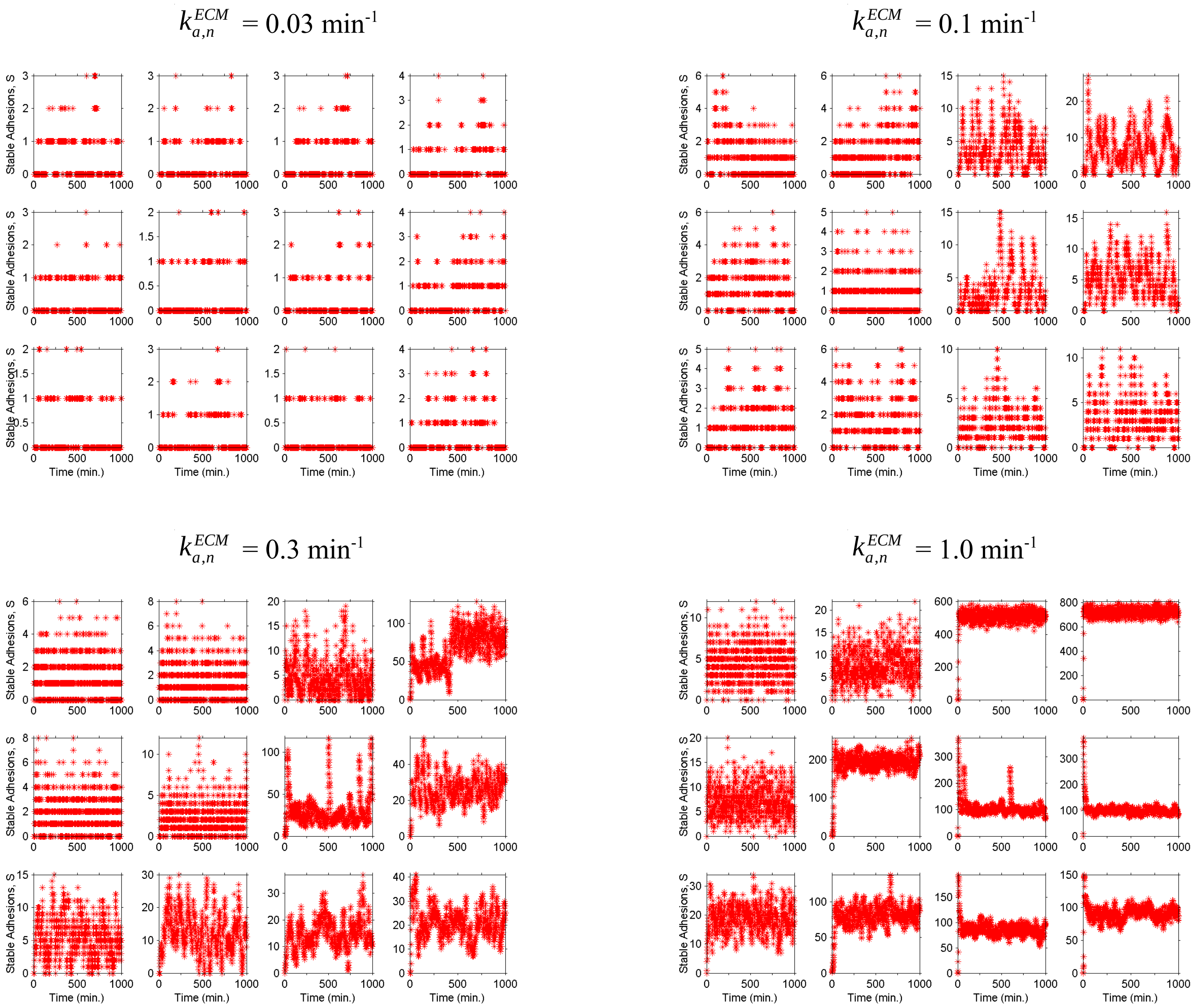

Supplement: Figure S2 — Raw simulation results corresponding to the analysis shown in Fig. 5 b. Stable adhesion number S is plotted as a function of time for the indicated values of and N* and a matrix of Es and In values as indicated in Fig. 5 a. (0.63 MB PDF) [file pcbi.1000688.s004.pdf]
