# Supplementary figures and images for: Stochastic Model of Integrin-Mediated Signaling and Adhesion Dynamics at the Leading Edges of Migrating Cells
Source: PLoS Comput Biol. 2010 Feb 26;6(2):e1000688. doi: 10.1371/journal.pcbi.1000688 (PMC2829041; doi:10.1371/journal.pcbi.1000688)

Fig. S3 A.  $I_n = 0$

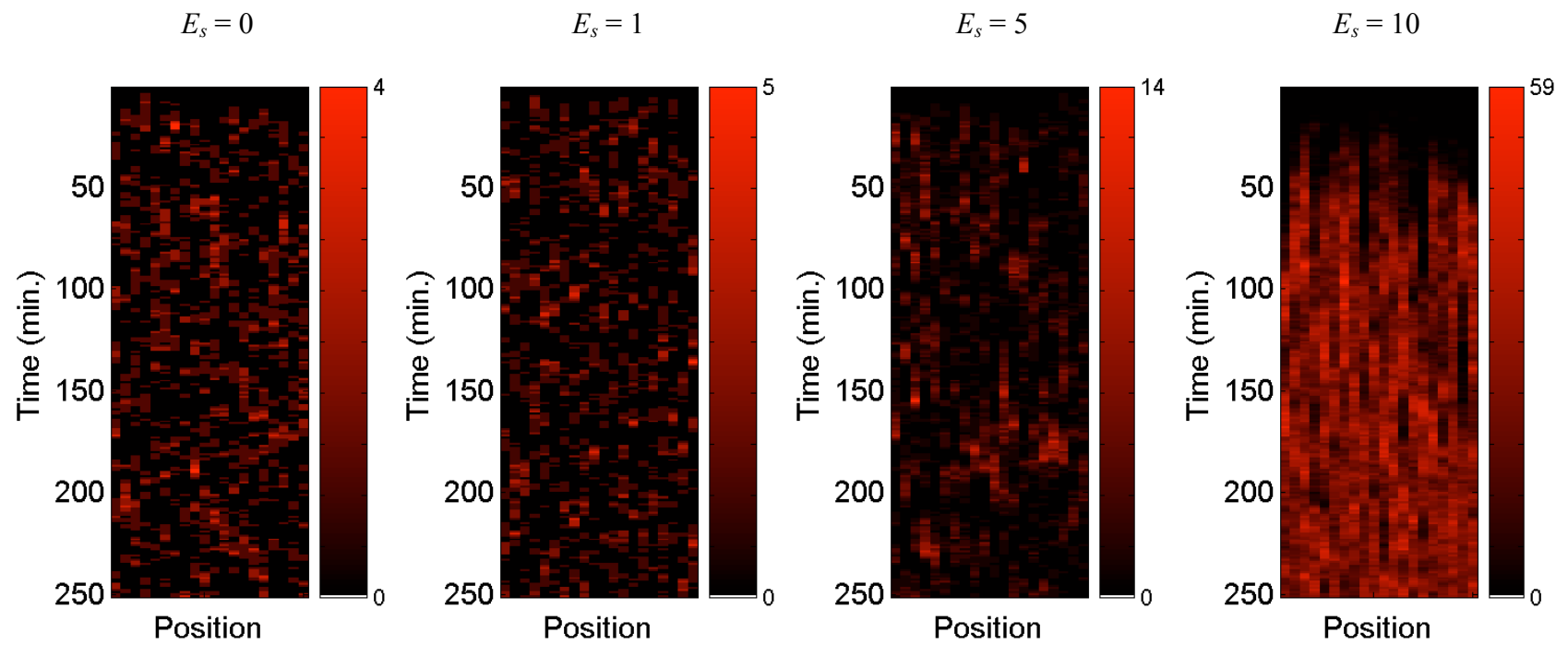

Fig. S3 B.  $I_n = 1$

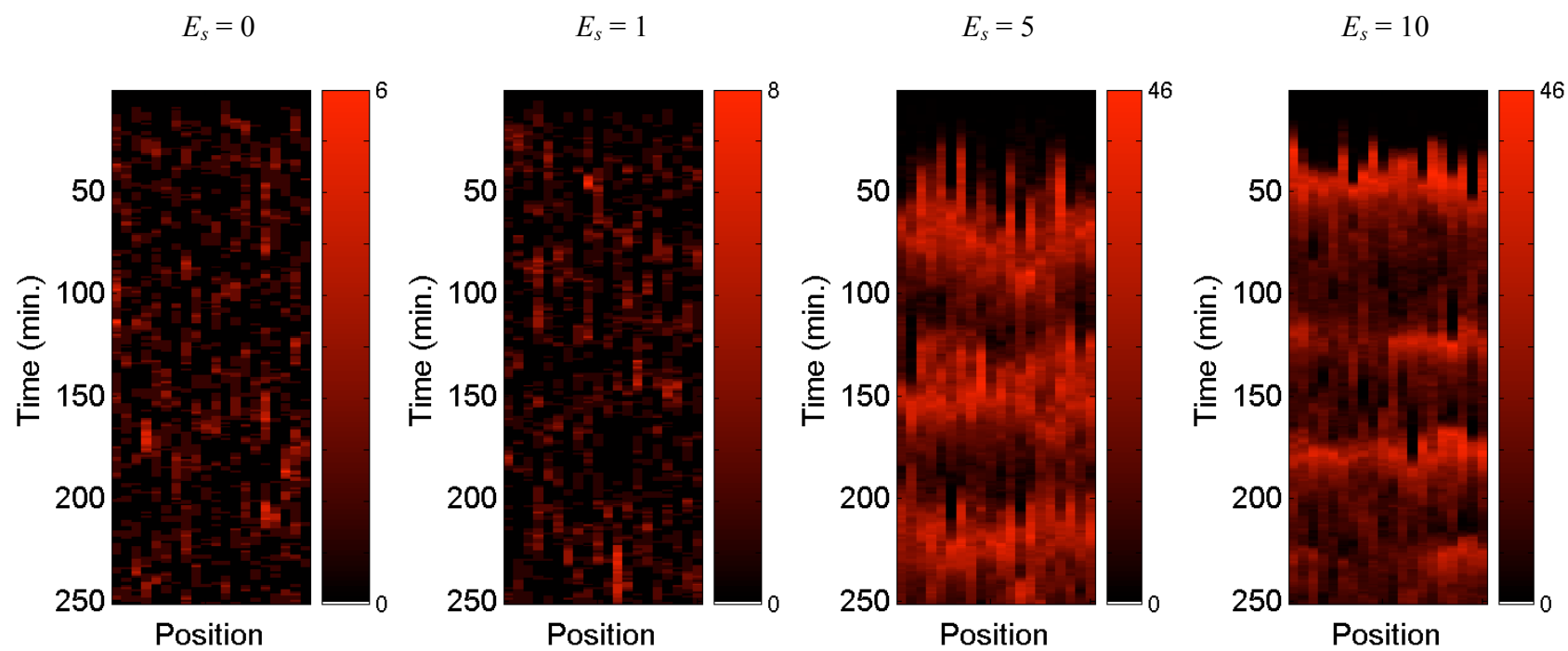

Fig. S3 C.  $I_n = 10$

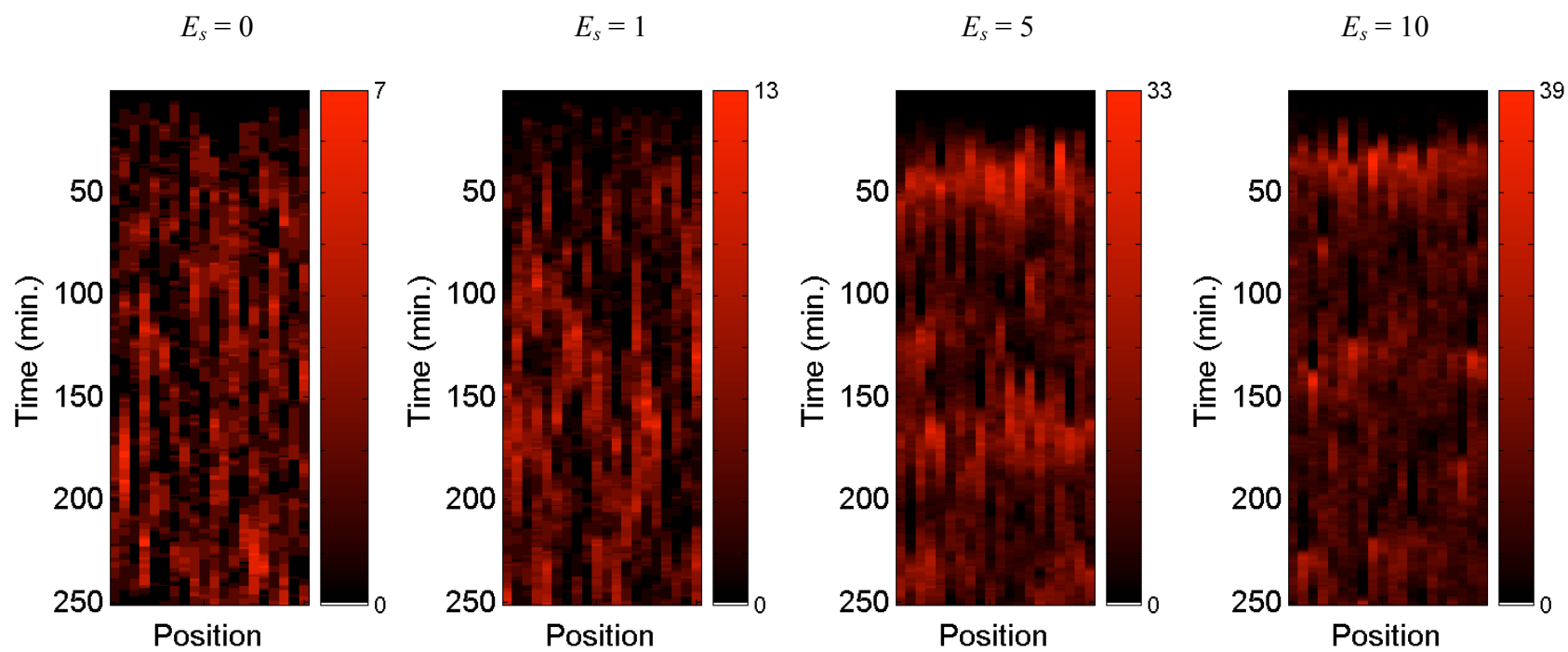

Supplement: Figure S3 — Spatially extended simulations were performed using the Next Subvolume Method, accounting for lateral diffusion of active Rac. Stable adhesion number S is indicated by the color scale as shown (red: S = maximum; black: S = 0) as a function of time and position. These results are from the same simulations used to generate the protrusion velocity results shown in Fig. 6. (0.30 MB PDF) [file pcbi.1000688.s005.pdf]
