# Supplementary material for: Stochastic Model of Integrin-Mediated Signaling and Adhesion Dynamics at the Leading Edges of Migrating Cells
Source: PLoS Comput Biol. 2010 Feb 26;6(2):e1000688. doi: 10.1371/journal.pcbi.1000688 (PMC2829041; doi:10.1371/journal.pcbi.1000688)
Supplement: Figure S4 — The velocity maps in Fig. 7 are shown in segmented form here. Red regions: velocity (nm/s) >5.0; yellow regions: 1.0< velocity (nm/s) <5.0 and contacting a red region; white regions: 1.0< velocity (nm/s) <5.0 but not contacting a red region; black regions: velocity (nm/s) <1.0. The contiguous red/yellow regions are considered bona fide protrusions. (0.28 MB PDF) [file pcbi.1000688.s006.pdf]

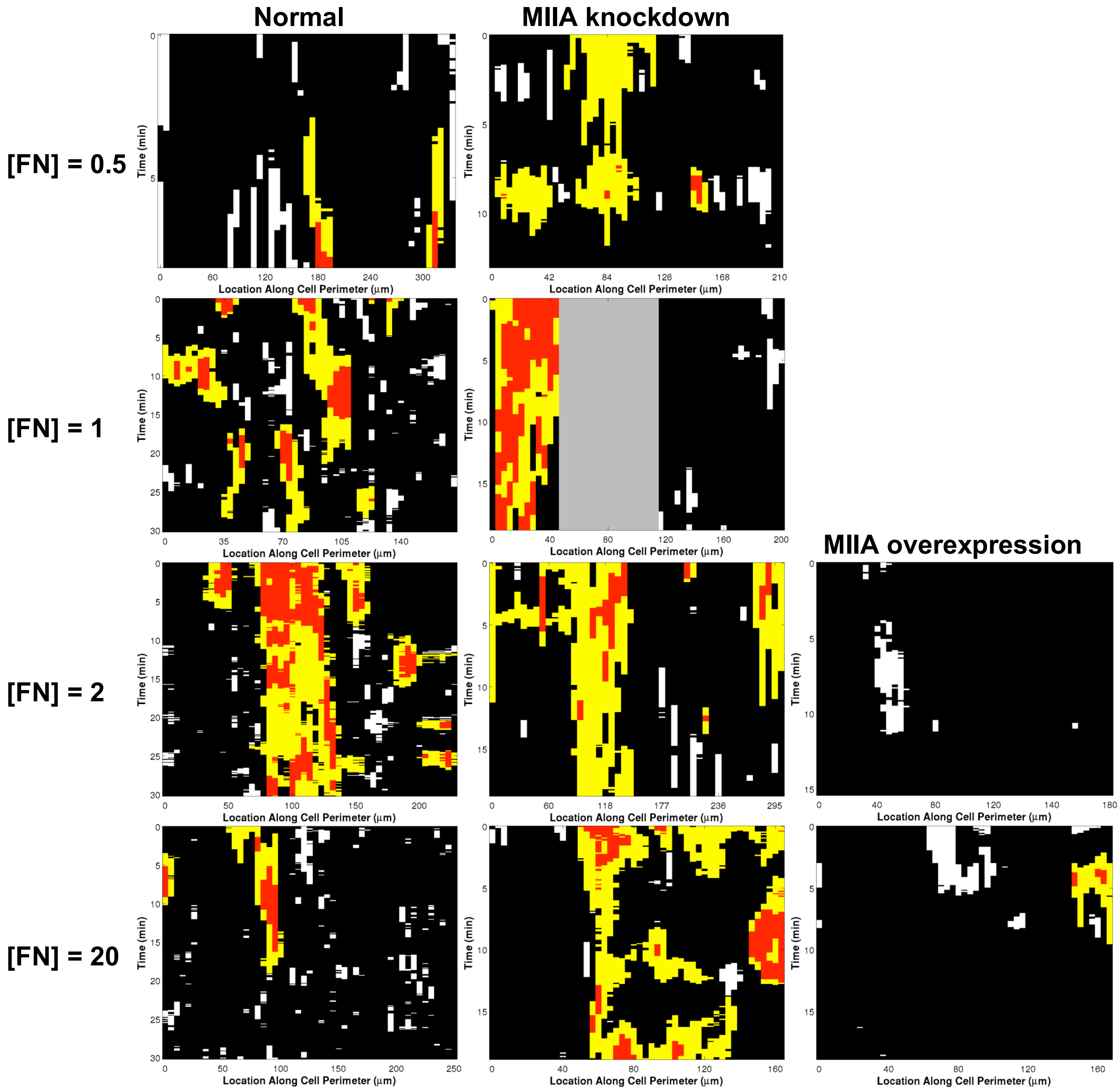

Figure S4. The velocity maps in Fig. 7 are shown in segmented form here. Red regions: velocity (nm/s) > 5.0; yellow regions: 1.0 < velocity (nm/s) < 5.0 and contacting a red region; white regions: 1.0 < velocity (nm/s) < 5.0 but not contacting a red region; black regions: velocity (nm/s) < 1.0. The contiguous red/yellow regions are considered *bona fide* protrusions.
